# A cross-sectional evidence-based review of pharmaceutical promotional marketing brochures and their underlying studies: Is what they tell us important and true?

**DOI:** 10.1186/1471-2296-7-13

**Published:** 2006-03-03

**Authors:** Roberto Cardarelli, John C Licciardone, Lockwood G Taylor

**Affiliations:** 1 Department of Family Medicine/Center for Evidence-Based Medicine, University of North Texas Health Science Center, Fort Worth, Texas, USA; 2 Osteopathic Research Center, University of North Texas Health Science Center, Fort Worth, Texas, USA; 3 Department of Family Medicine/Center for Evidence-Based Medicine, University of North Texas Health Science Center, Fort Worth, Texas, USA

## Abstract

**Background:**

A major marketing technique used by pharmaceutical companies is direct-to-physician marketing. This form of marketing frequently employs promotional marketing brochures, based on clinical research, which may influence how a physician prescribes medicines. This study's objective was to investigate whether or not the information in promotional brochures presented to physicians by pharmaceutical representatives is accurate, consistent, and valid with respect to the actual studies upon which the promotional brochures are based.

**Methods:**

Physicians in five clinics were asked to consecutively collect pharmaceutical promotional brochures and to send them all to a centralized location. The brochures for any class of medication were collected on a continuous basis until 20 distinct promotional brochures were received by a central location. Once the brochure was received, the corresponding original study was obtained. Two blinded reviewers performed an evidence-based review of the article, comparing data that was printed on the brochure to what was found in the original study.

**Results:**

Among the 20 studies, 75% of the studies were found to be valid, 80% were funded by the pharmaceutical company, 60% of the studies and the corresponding brochures presented patient-oriented outcomes, and 40% were compared to another treatment regimen. Of the 19 brochures that presented the data as graphs, 4 brochures presented a relative risk reduction while only 1 brochure presented an absolute risk reduction. 15% of the promotional marketing brochures presented data that was different from what was in the original published study.

**Conclusion:**

Given the present findings, physicians should be cautious about drawing conclusions regarding a medication based on the marketing brochures provided by pharmaceutical companies.

## Background

The pharmaceutical industry spent over $11 billion in pharmaceutical marketing, excluding medication samples, in 2004, with more than $7 billion directed to clinicians [1]. This creates the potential for an ethical dilemma because such marketing may influence physician prescribing behavior without necessarily benefiting the patient [2-6]. Such marketing also creates the potential for inappropriate prescribing practices, which contribute to escalating national health care costs[7].

There is an ongoing debate on the effects of pharmaceutical marketing on health care delivery. For example, while diuretic and beta-blocker medications are considered the first line antihypertensive therapies (both medications being relatively inexpensive and available in generic forms), extensive marketing campaigns by pharmaceutical companies to promote calcium channel blockers (CCBs) resulted in the largest growth in revenues for this class of anti-hypertension drugs during the 1990s [8]. The percentage of CCB advertisements increased from 4.6% in 1986 to 26.9% in 1996, while advertisements for beta-blockers and diuretics decreased from 12.4% to 0% and from 4.2% to 0%, respectively, during the same time period [9]. It was also noted that CCBs accounted for 38% of antihypertensive prescriptions written in 1995, while diuretics and beta-blockers combined accounted for only 19% [8]. This, in essence, affected the standard of care in the management of hypertension, [10] even though CCBs may increase the risk of fatal and nonfatal myocardial infarction by 18% [11]. Lack of compliance with antihypertensive guidelines, by using second-line medications over first-line medications, was associated with potential increases in health care expenditures in the range of $2.6 billion to $3.2 billion in 1996 [7].

Direct-to-physician (DTP) marketing is one important facet of the promotion of pharmaceuticals. DTP includes verbal in-office presentations which are usually accompanied by promotional advertising brochures, free medication samples, and possibly gifts such as meals or other promotional items. These in-office presentations by pharmaceutical representatives (PRs) are an important method of promotion for pharmaceutical companies. Their impact is undeniable, as PRs are most frequently reported by physicians as their source of primary information about new medications [12]. While direct-to consumer (DTC) pharmaceutical marketing has increased dramatically in the last decade, DTP marketing expenditures remain 48% greater than for DTC marketing [1].

There is concern about the influence of DTP marketing on physician prescribing practices and its consequences, such as the physician's ethical obligation to the patient and health care costs. Studies have repeatedly shown that pharmaceutical promotion influences physician behavior [3,12-14]. The more physicians are encountered by PRs, the more likely they are to deny that they are influenced by pharmaceutical gifts and PR interactions [15]. Some information provided by PRs to physicians has been found to be misleading or biased [5,16-21]. Eleven percent of the verbal statements about drugs made by pharmaceutical representatives to physicians has been found to be inaccurate [16]. Further, a majority of journal advertisements have been found to be based on studies of poor methodological quality [22].

While studies have investigated the accuracy and influence of promotional information provided by PRs to physicians, [3,5,8,12-14,16-21] no study has assessed the visual nature and accuracy of the data that are presented in the promotional brochures. The accuracy and validity of the visual displays and data presented in promotional brochures is important because physicians who rely more on promotional information tend to be heavier prescribers and more willing to try new medications [13,23-31]. The objective of this study was to review the data that is visually presented on advertisement brochures for its accuracy, consistency, and validity.

## Methods

The study was conducted in 5 family medicine clinics of the University of North Texas Health Science Center at Fort Worth. This study was exempt from a formal Institutional Review Board review. Physicians in these clinics were asked to collect promotional brochures that accompanied the PR's verbal presentation and to indicate the one figure, table, or data that was emphasized most by the PR. This process was used because we believed that the data emphasized by the PR would have the greatest impact on changing a physician's behavior. A convenience sample of 20 distinct brochures was collected between October 15, 2004 and December 5, 2004. Each brochure represented one medication, outcome, and its respective study. The sample size was determined *a priori *because no studies were available to calculate an accurate sample size. The underlying cited publication on the brochure in support of the identified promotional claim was obtained for review. Two reviewers used a standardized form to collect data and perform an evidence-based review of the article, comparing data that were presented on the brochure to those found in the underlying study. Both reviewers were initially blinded to each other's review. The validity of an article was determined by a series of six questions that addressed evidence-based principles (Table 1) [32]. Validity was determined by the following method: 3 of 3 major criteria and at least 2 of 3 minor criteria must have been met. The disclaimers and acknowledgments of the original study were reviewed to determine whether a pharmaceutical company funded the study. The outcome of interest on the brochure was classified as patient-oriented or disease-oriented. Patient-oriented outcomes were those that directly affected or reflected the patient, such as mortality or change in perceived symptoms such as urinary frequency or pain. Disease-oriented outcomes were those characteristics of the disease itself, such as change in blood pressure measurements. The brochures' data and their corresponding studies were reviewed to determine if the therapy (i.e. medication) was compared to another therapy, placebo, or neither. The reviewers also assessed whether relative risk changes (reduction or increase), absolute risk changes, or both were presented on the brochures, including any corresponding visual graphic. In addition, the reviewers visually assessed how these data were graphically presented on the brochures, such as the use of charts, arrows, or line graphs. The statistical significance of the outcome of interest was also assessed from both sources. The reviewers compared the data presented on the brochure to the data presented in the original study to determine consistency. The results were entered into a database and any discrepancies between the reviewers were subsequently resolved by consensus. Data management and statistical analysis were performed using the SPSS software [33].

**Table 1:** Validity measures

**Major criteria**
1. Was the assignment of patients to treatments randomized?
2. Were all patients who entered the trial accounted for at its conclusion?
3. Were the patients analyzed in the groups to which they were randomized?
**Minor criteria**

1. Were patients and clinicians kept "blind" to which treatment was being received?
2. Aside from the experimental treatment, were the groups treated equally?
3. Were the groups similar at the start of the trial?

A Cohen's kappa and a prevalence-adjusted bias-adjusted kappa (PABAK) [34] were calculated on validity assessments to obtain an index of inter-rater reliability. While under most circumstances a Cohen's kappa value would suffice, we believe that both prevalence (the unequal distribution of "Yes" vs. "No" validity decisions between the reviewers) and bias (from the reviewers' differing assessments of the frequency of study validity) may have yielded misleading results given our high percentage of crude agreement. This concept has been explained in greater detail by Feinstein and Cicchetti[35] and Byrt et al. [34]. Thus a PABAK value may better measure agreement between the two reviewers as it adjusts for factors (prevalence, bias, and agreement) present in an unbalanced 2 × 2 agreement table.

## Results

The characteristics of the 20 brochures are presented in Table 2. The greatest number of brochures described cardiovascular medications (45%), followed by neurological medications (15%), genito-urinary medications (10%), chronic pain medications (10%), diabetes mellitus medications (5%), gastrointestinal medications (5%), infections disease medications (5%), and osteoporosis medications (5%). The reviewers rated 75% of the studies as valid. Among the 20 underlying studies, 80% were funded by the pharmaceutical company, 5% (1 study) was not funded by the pharmaceutical industry, and 15% were of unspecified funding. Sixty percent of the underlying studies and their corresponding brochures presented patient-oriented outcomes, such as mortality, pain scores, and episodes of urinary incontinence. Only 8 of the 20 (40%) medications were compared to another treatment regimen. Four brochures presented relative risk reduction (RRR), 1 presented absolute risk reduction (ARR), and 15 presented some other statistic, such as a mean change from baseline. A total of 95% of these brochures used graphics to emphasize the differences between groups, such as bar charts, arrows, or line graphs. Of these graphs, 21% presented RRR, 5% presented ARR, and 74% presented some other statistic. Only 1 brochure presented data that were not statistically significant. Three of the twenty (15%) brochures presented data that were different from those published in the underlying studies.

**Table 2:** Study measures

	(N = 20)	
	n	(%)
1. Was the study valid?		
Yes	15	(75)
No	5	(25)
2. Was the study funded by a pharmaceutical company?		
Yes	16	(80)
No	1	(5)
Unknown	3	(15)
3. What was the study outcome?		
Patient-oriented	12	(60)
Disease-oriented	8	(40)
4. What was the study therapy compared to?		
Another medication or therapy	8	(40)
Placebo	10	(50)
Neither	2	(10)
5. Was a graphic* used on the brochure?		
Yes	19	(95)
No	1	(5)
6. Of the brochures with graphics, how was the data presented?		
Relative risk reduction	4	(21)
Absolute risk reduction	1	(5)
Other**	14	(74)
7. Was the outcome of interest statistically significant?***		
Yes	19	(95)
No	1	(5)
8. Did the data on the brochure differ from the underlying study?		
Yes	3	(15)
No	17	(85)

*Includes bar charts, line graphs, pie charts, arrows

**Includes mean change, point differences, etc

***p < 0.05

A value of .41 was calculated for Cohen's kappa, and the PABAK value was .60, indicating a moderate level of agreement between the two reviewers [36]. The crude level of overall agreement, unadjusted for chance, was 80%.

## Discussion

Seventy five percent of the reviewed articles were considered valid. This supports findings from a previous study that assessed whether the quality of a study was influenced by sponsorship [37]. The authors found that the majority of the studies sponsored by pharmaceutical companies were of good quality. However, they also found that published studies that were sponsored by pharmaceutical companies were four times more likely to have outcomes favoring the sponsor's product than were studies that had other types of sponsors. One potential solution to reducing the skepticism resulting from industry-funded research is to have pharmaceutical companies improve access to their original data. Another possible solution is for academia to have funding available to independently conduct studies without the influence of the pharmaceutical companies. Although pharmaceutical companies exert control over their clinical trials, clinical outcome studies conducted at academic health science centers may have a role in complementing the post-marketing surveillance of new drugs following FDA approval.

A large majority of the brochures presented data that were based on underlying studies funded by the pharmaceutical company. However, a recent study by Cooper and Schriger found that only 58% of the original research cited in advertisements was sponsored by a pharmaceutical company or had a company affiliated author [38]. This deviation from our results may be due to differences in methods for ascertaining funding source and product mix analyzed (e.g., brochures vs journal advertisements). Alternatively, it may also reflect our study's small sample size. There has been concern that industry-supported manuscripts may selectively report study data that make a medication or therapy appear more efficacious than it actually is [39-41]. The clinician's dilemma becomes, "Are the results from this 'valid study' truly valid?"

Although a majority of the outcomes in the reviewed studies were patient-oriented, a substantial number were not. It is important to present data that reflects the outcome of the patient, such as mortality, quality of life changes, and symptom reduction. Thus, while a CCB may reduce blood pressure just as effectively as a diuretic, patient outcomes (e.g., mortality) may prevail in the long term [11,42].

While 95% of the brochures presented a visual graphic, only 1 brochure presented an absolute risk reduction and 4 brochures presented relative risk reductions. This "framing effect" may mislead physicians to conclude that a large difference in outcome occurs with the use of the promoted medication or therapy [32]. Moreover, as the consistency of outcomes was being assessed in the underlying study and its subsequent promotional brochure, the reviewers noted that the overwhelming majority of data selected for the brochure were based on a desired visual impact. Although it was not a part of this study's objectives, it appeared that pharmaceutical companies often selected the outcome with the greatest relative risk reduction or percent-change from baseline in lieu of more clinically important findings in the original manuscript. This is an area for further research and scrutiny.

A minority of the studies (eight of 20) compared the medication to another medication or treatment strategy. When a medication has already been approved by the Food and Drug Administration, subsequent studies that compare that medication to placebo have limited value to practicing physicians, as efficacy has already been established. It is of more value to physicians for studies to compare new medications to generic and inexpensive medications that are currently used in practice to determine if a change in disease management is indicated.

Although three of the 20 brochures presented data that were different from the original study, the differences were small and most likely would not have affected the clinician's prescribing behavior.

This study had several limitations. Only two blinded reviewers were available and any discrepancies were resolved by consensus rather than adjudication by a third reviewer. The method in which the reviewers deemed the underlying studies valid has not been validated in previous studies. The sample size may have been too small to detect clinically important findings. Because brochures were collected only in selected family medicine clinics in one city, these findings may not be generalized to other specialties, settings (i.e. hospital), or geographic regions. Only brochures were studied, therefore these findings may not be generalized to other types of promotional products or advertisements, such as journal advertisements. We believe that successful drug marketing to physicians entails more than simply presenting a brochure to the physician and this study only assessed one aspect of drug marketing tactics. There may be seasonal variations in drug marketing that further limit generalizability, although this may have been mitigated to some degree by the wide range of drugs included in the study.

## Conclusion

Given the present findings, physicians should be cautious about drawing conclusions based on data presented on brochures provided by pharmaceutical companies. It would be prudent for physicians to review the original study prior to changing prescribing behavior based on promotional brochures only. Further, physicians should be familiar with and utilize the principles of evidence-based medicine in assessing the validity of published studies. As our study was a descriptive cross-sectional study with the primary purpose of using a semi-objective methodology to review pharmaceutical brochures and their corresponding published studies, future research needs to determine not only how the promotional brochure plays in overall DTP marketing and how pharmaceutical brochures affect physician prescribing behaviors, but also if patient outcomes are associated with such changes.

## Competing interests

The author(s) declare that they have no competing interests.

## Authors' contributions

RC, JCL, and LGT designed and implemented the study. RC and JCL reviewed the brochures and their underlying studies to assess for validity. RC, JCL, and LGT conducted the analyses and drafted the manuscript.

## Pre-publication history

The pre-publication history for this paper can be accessed here:


